# Acellular Bi-Layer Silk Fibroin Scaffolds Support Tissue Regeneration in a Rabbit Model of Onlay Urethroplasty

**DOI:** 10.1371/journal.pone.0091592

**Published:** 2014-03-14

**Authors:** Yeun Goo Chung, Duong Tu, Debra Franck, Eun Seok Gil, Khalid Algarrahi, Rosalyn M. Adam, David L. Kaplan, Carlos R. Estrada Jr., Joshua R. Mauney

**Affiliations:** 1 Department of Urology, Urological Diseases Research Center, Boston Children's Hospital, Boston, Massachusetts, United States of America; 2 Department of Surgery, Harvard Medical School, Boston, Massachusetts, United States of America; 3 Department of Biomedical Engineering, Tufts University, Medford, Massachusetts, United States of America; Université de Technologie de Compiègne, France

## Abstract

Acellular scaffolds derived from *Bombyx mori* silk fibroin were investigated for their ability to support functional tissue regeneration in a rabbit model of urethra repair. A bi-layer silk fibroin matrix was fabricated by a solvent-casting/salt leaching process in combination with silk fibroin film casting to generate porous foams buttressed by homogeneous silk fibroin films. Ventral onlay urethroplasty was performed with silk fibroin grafts (Group 1, N = 4) (Width×Length, 1×2 cm^2^) in adult male rabbits for 3 m of implantation. Parallel control groups consisted of animals receiving small intestinal submucosa (SIS) implants (Group 2, N = 4) or urethrotomy alone (Group 3, N = 3). Animals in all groups exhibited 100% survival prior to scheduled euthanasia and achieved voluntary voiding following 7 d of initial catheterization. Retrograde urethrography of each implant group at 3 m post-op revealed wide urethral calibers and preservation of organ continuity similar to pre-operative and urethrotomy controls with no evidence of contrast extravasation, strictures, fistulas, or stone formation. Histological (hematoxylin and eosin and Masson's trichrome), immunohistochemical, and histomorphometric analyses demonstrated that both silk fibroin and SIS scaffolds promoted similar extents of smooth muscle and epithelial tissue regeneration throughout the original defect sites with prominent contractile protein (α-smooth muscle actin and SM22α) and cytokeratin expression, respectively. *De novo* innervation and vascularization were also evident in all regenerated tissues indicated by synaptophysin-positive neuronal cells and vessels lined with CD31 expressing endothelial cells. Following 3 m post-op, minimal acute inflammatory reactions were elicited by silk fibroin scaffolds characterized by the presence of eosinophil granulocytes while SIS matrices promoted chronic inflammatory responses indicated by mobilization of mononuclear cell infiltrates. The results of this study demonstrate that bi-layer silk fibroin scaffolds represent promising biomaterials for onlay urethroplasty, capable of promoting similar degrees of tissue regeneration in comparison to conventional SIS scaffolds, but with reduced immunogenicity.

## Introduction

The urethra serves as a crucial outlet conduit through which urine is expelled from the urinary tract. It has a major role in the urinary continence mechanism [1], and in the adult male, an intact urethra is essential for the adequate anterograde transport of seminal fluid and ultimately fertility [2]. A wide variety of congenital and acquired pathologies including hypospadias, epispadias, strictures, fistulas, malignancy, and straddle injuries can compromise the normal functionality of the urethra necessitating organ reconstruction [3]–[7]. End-to-end anastomosis is frequently used to repair short, non complex urethral defects wherein organ continuity is surgically restored by aligning and joining normal tissue segments [8]. In circumstances in which there is a lack of patient urethral tissue, extragenital skin flaps [9], [10], buccal mucosa [11], bladder mucosa [12], [13], and tunica vaginalis [14] have been utilized clinically as autologous tissue grafts for urethroplasty procedures. In addition to the risk of donor site morbidity, the long-term success of these implants is often suboptimal due to significant complications such as fistula formation [15], recurrent strictures [16], hair growth [17], stone formation [18], diverticula [19], and meatal stenosis [20]. Given the limitations associated with conventional surgical approaches, there exists a substantial need for the development of alternative strategies for urethral tissue replacement.

Over the past several decades, an array of natural and synthetic, biodegradable scaffolds either alone or seeded with ex vivo expanded, primary cell sources have been investigated for urethral tissue engineering applications [21], [22]. Decellularized collagen-based biomaterials such as small intestinal submucosa (SIS) and bladder acellular matrix (BAM) have shown promise as acellular grafts for onlay urethroplasty. These matrices have been demonstrated to promote epithelial and smooth muscle tissue regeneration in short, semi-circumferential urethral defects by supporting host tissue integration in both animal models [23]–[25] as well as patients afflicted with hypospadias [26] and urethral strictures [27], [28]. Although initial defect consolidation is often achieved, long-term adverse side-effects such as stricture recurrence and fistula formation have been reported [29], [30], therefore raising the risk for graft failure and the need for surgical re-intervention.

Synthetic biomaterials derived from polyesters have also been explored for urethral substitution [31]–[33]. In particular, a recent study by the Atala group demonstrated the efficacy of tubularized constructs composed of poly-glycolic acid: poly-(lactide-co-glycolic acid) meshes seeded with autologous bladder cells for treatment of traumatic urethral injuries in children [33]. Short-term results revealed this approach achieved regeneration of native tissue architecture as well as restoration of organ function in the majority of study participants. However, degradation metabolites of polyester-based scaffolds are known to elicit chronic inflammatory responses *in vivo* [34] and therefore have the potential to negatively impact long-term organ function due to adverse foreign body reactions [35]. In addition, the requirement for invasive tissue biopsies and the costs associated with ex vivo cell propagation for implant seeding still remain practical barriers for widespread clinical utilization of this technology [22]. We hypothesized that a useful strategy for urethral tissue engineering would consist of an “off the shelf” acellular graft with structural, mechanical, and degradation properties sufficient to support initial defect stabilization and organ continuity while allowing for gradual remodeling and host tissue regeneration without deleterious immunogenic reactions.

Silk fibroin scaffolds derived from *Bombyx mori* silkworm cocoons represent versatile platforms for urogenital tissue reconstruction due their mechanical robustness [36], diverse processing plasticity [37], and tailorable biodegradability [35], [38]. Previous reports have demonstrated the ability of acellular silk fibroin matrices to support the formation of innervated, vascularized smooth muscle and urothelial tissues in both rodent and porcine models of bladder augmentation [39], [40]. In vivo comparisons between conventional SIS and poly-glycolic acid-based biomaterials have also shown that silk fibroin scaffolds offer distinct advantages for bladder tissue repair including improved functional organ performance, reduced inflammatory reactions, and enhanced tissue regeneration [39], [41]. In the current study, we evaluated the efficacy of an acellular, bi-layer silk fibroin matrix to mediate tissue regeneration in a rabbit model of onlay urethroplasty. The bi-layer scaffold configuration is composed of a porous silk fibroin foam which serves to allow for ingrowth of surrounding host tissues while an annealed silk fibroin film functions to provide a fluid-tight seal for retention of hollow organ contents (i.e. urine) during defect consolidation [39], [40]. Our group has recently demonstrated that bi-layer silk fibroin matrices support superior functional tissue regeneration in comparison to multi-laminate gel spun silk fibroin biomaterials in a rat model of bladder tissue repair [39]. Therefore, the utility of this bi-layer matrix for urethral tissue engineering was investigated.

## Materials and Methods

### Ethics Statement

*B. mori* silkworm cocoons were obtained from a commercial supplier (Tajima Shoji Co., Yokohama, Japan) and therefore no specific field studies were performed for their acquisition. This study was carried out in strict accordance with the recommendations in the Guide for the Care and Use of Laboratory Animals of the National Institutes of Health. All animal experiments were performed with approval by Boston Children's Hospital Animal Care and Use Committee (Protocol Number: 12-06-2196). All surgery was performed under isoflurane anesthesia, and all efforts were made to minimize suffering.

### Biomaterials

Aqueous silk fibroin solutions were prepared from *B. mori* silkworm cocoons using published procedures [36] and utilized to construct a bi-layer silk fibroin matrix using methods previously described [39]. Briefly, a silk fibroin solution (8% wt/vol) was poured into a rectangular casting vessel and dried in a laminar flow hood at room temperature for 48 h to achieve formation of a silk fibroin film. A 6% wt/vol silk fibroin solution was then mixed with sieved granular NaCl (500–600 µm, average crystal size) in a ratio of 2 g NaCl per ml of silk fibroin solution and layered on to the surface of the silk fibroin film. The resultant solution was allowed to cast and fuse to the silk fibroin film for 48 h at 37°C and NaCl was subsequently removed by washing the scaffold for 72 h in distilled water with regular volume changes. Bi-layer silk fibroin scaffold morphology was assessed by scanning electron microscopy using published protocols [40]. Before implantation, silk fibroin scaffolds were sterilized in 70% ethanol and rinsed in phosphate buffered saline (PBS) overnight. SIS grafts (Cook, Bloomington, IN) were evaluated in parallel as a standard point of comparison. Tensile properties of both scaffold configurations have been previously reported [39].

### Surgical Procedures

Scaffold groups (Silk fibroin: N = 4; SIS: N = 4) were evaluated in a ventral onlay urethroplasty model [42] using adult male New Zealand white rabbits (3–3.5 kg, ∼3–4 m of age). Surgery was performed under sterile technique with maintenance anesthesia of 2–3% isoflurane following induction with intramuscular injection of 0.04 mg/kg glycopyrrolate. All rabbits were kept under mechanical ventilation for the duration of the operative procedures. Following urethral catheterization, the penile urethra was exposed through a ventral midline skin incision and mobilized from the underlying corpora spongiosum. A 1×2 cm^2^ (Width×Length) area of ventral urethral tissue was excised and a biomaterial graft of equal size was anastomosed to the defect site using interrupted 6-0 polyglactin sutures. Non absorbable 6-0 polypropylene sutures were placed at the proximal, distal, and lateral boundaries of the implantation area for identification of graft borders. Skin incisions were subsequently closed with running sutures. In addition, a control group of animals (N = 3) receiving urethrotomy alone was treated similarly in parallel. All animals were administered prophylactic antibiotics (5 mg/kg/d Baytril®) for 3 d post-operatively. For all experimental groups, an 8 French Firlit-Kluge urethral stent (Cook Urological, Spencer, IN) was secured to the urethra to allow for reinforcement of the repair site and free urine drainage via catheterization for 7 d following surgical procedures. After stent removal, animals were allowed to void voluntarily until the completion of the study. Analysis of serum creatinine levels was performed in all animals pre-operatively and at 1 and 3 m post-operatively. Controls and rabbits receiving implants were euthanized at 3 m post-implantation and isolated urethral specimens were subjected to histological, immunohistochemical, and histomorphometric analyses.

### Retrograde urethrography (RUG)

Retrograde urethrography was performed pre-operatively and at 3 m post-repair in all experimental groups to evaluate regenerated urethra continuity and the presence of strictures, fistulas or stone formation as previously described [42]. Under general anesthesia, an 8 French feeding tube was inserted into the urethral meatus and 1.5 ml iothalamate meglumine 17.2% (Cysto-Conray II, Mallinckrodt Inc, St. Louis MO) saline solution was injected in a retrograde fashion through the feeding tube while X-rays were taken in the supine and lateral positions.

### Histological, immunohistochemical, and histomorphometric analyses

Regenerated and control tissue segments from the proximal, distal and central regions of the original surgical site were excised for standard histological processing. Briefly, specimens were fixed in 10% neutral-buffered formalin, dehydrated in graded alcohols, and then embedded in paraffin. Sections (5 µm) were cut and then stained with hematoxylin and eosin (H&E) or Masson's trichrome (MTS) using routine histological protocols. For immunohistochemical (IHC) analyses, contractile smooth muscle markers such as α-smooth muscle actin (α-SMA) and SM22α; epithelial-associated proteins, cytokeratins (CK); neuronal and endothelial markers, synaptophysin (SYP38) and CD31, respectively, were detected using the following primary antibodies: anti-α-SMA [Sigma-Aldrich, St. Louis, MO, 1∶200 dilution], anti-SM22α [Abcam, Cambridge, MA, 1∶200 dilution], anti-pan-CK [Dako, Carpinteria, CA, 1∶200 dilution], anti-SYP38 [Abcam, 1∶200 dilution], anti-CD31 [Abcam, 1∶100 dilution]. Sections were then incubated with species-matched Cy3-conjugated secondary antibodies (Millipore, Billerica, MA) and nuclei were counterstained with 4′, 6-diamidino-2-phenyllindole (DAPI). Specimens were visualized using an Axioplan-2 microscope (Carl Zeiss MicroImaging, Thornwood, NY) and representative images were acquired using Axiovision software (version 4.8).

Histomorphometric analysis was performed to assess the degree of smooth muscle and epithelial tissue regeneration in both control and implant groups using ImageJ software (version 1.47). Image thresholding and area measurements were carried out on 12–18 independent microscopic fields (magnification 10×, α-SMA; 20×, CK) equally dispersed along the proximal, distal, and central regions of the original surgical sites to determine the percentage of tissue area occupied by α-SMA+ smooth muscle bundles and CK+epithelium relative to the total tissue area examined. In addition, the number and diameter of CD31+ vessels and aggregates of mononuclear cell infiltrates (>100 µm in diameter) were measured similarly in 6 independent microscopic fields (magnification 10×) and normalized to the total tissue area examined to ascertain the extent of *de novo* vascularization and chronic inflammatory reactions, respectively, in all experimental groups. Data for these measurements (N = 3–4 animals/group) were analyzed with the Kruskal-Wallis test in combination with the post-hoc Scheffé's method using SPSS Statistics software v19.0 (http://www.spss.com) and expressed as mean ± standard deviation. Statistically significant values were defined as *p*<0.05.

## Results and Discussion

**Figure 1:**
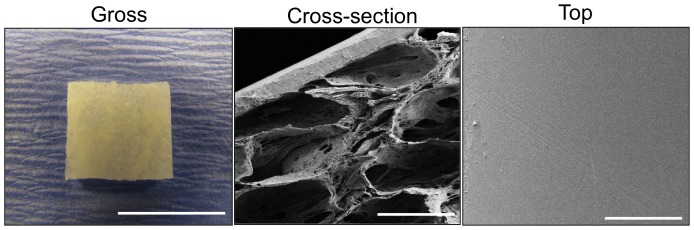
Structural characterization of silk fibroin scaffold. Photomicrographs of gross scaffold morphology (scale bar = 1 cm) and SEM images of cross-sectional and top views of bi-layer scaffold architecture (scale bars = 400 µm).

SEM analysis of the bi-layer silk fibroin matrix revealed the formation of distinct structural compartments (**Figure 1**). The solvent-cast/NaCl-leached layer comprised the bulk of the total matrix thickness (2 mm) and resembled a foam configuration with large pores (pore size, ∼400 µm) interconnected by a network of smaller pores dispersed along their periphery. This compartment was buttressed on the external face with a homogenous, non porous silk fibroin layer (200 µm thick) generated by film annealment during casting.

**Figure 2:**
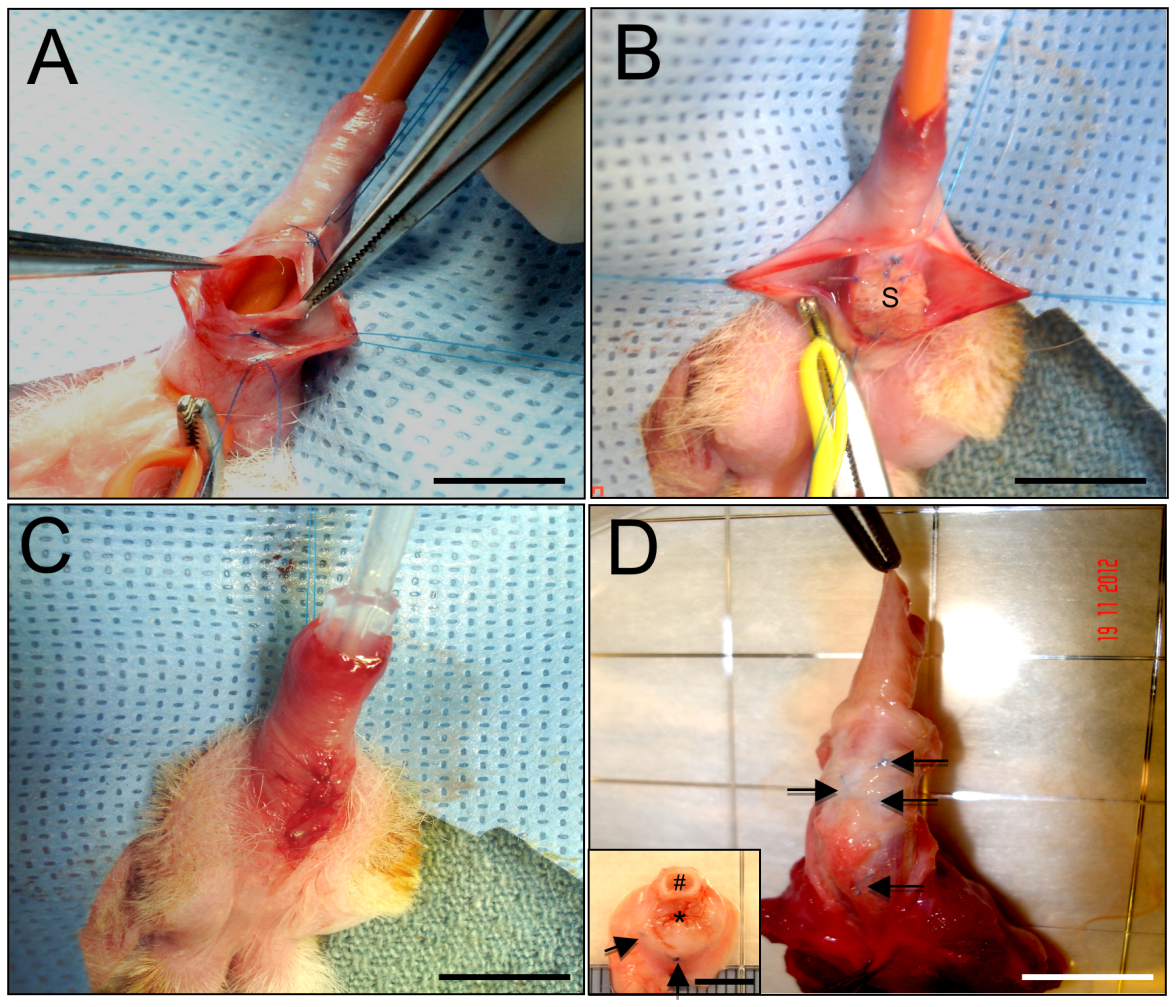
Ventral onlay urethroplasty model. Photomicrographs of various surgical stages of silk fibroin scaffold implantation and gross morphology of regenerated tissue. [A] Excision of native tissue and defect creation in penile urethra. [B] Anastomosis of silk fibroin graft (S) into the urethral defect. [C] Surgical closure of defect site. [D] Original implantation site following 3 m post-op. Inset: cross-section of harvested penis with corpora cavernosa (#) and regenerated urethra (*). Arrows denote marking sutures. [A–D], scale bar = 2 cm; inset, scale bar = 0.6 cm.

**Figure 3:**
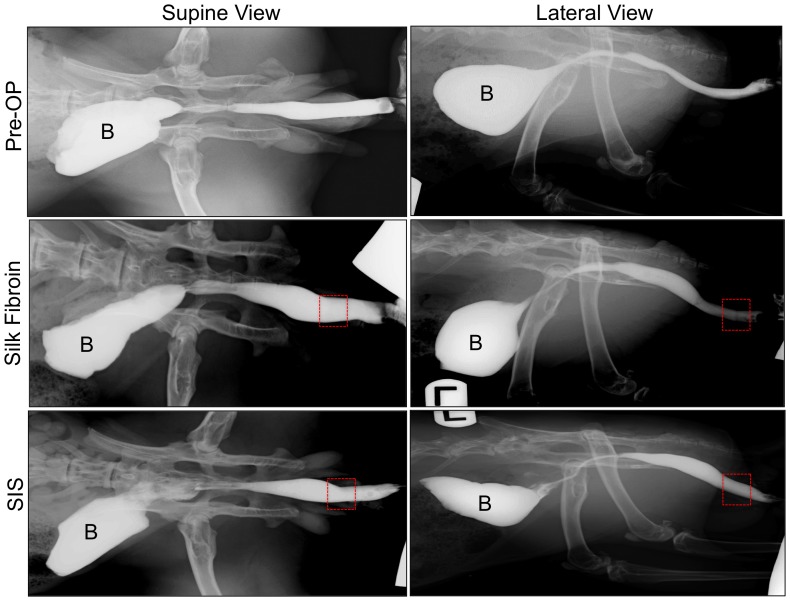
Retrograde urethrography in matrix-grafted animals at pre-op and following 3 m of implantation. Boxes denote original implantation sites while B denotes contrast-instilled bladders.

Ventral onlay urethroplasty was a feasible approach for surgical integration of both SIS and silk fibroin scaffolds into urethral defects (**Figure 2A–C**). Animals in each experimental group had an uneventful post-operative period with no mortality observed prior to scheduled euthanasia. Over the course of the 3 m implantation period, voluntary voiding was achieved in all animals studied following initial 7 d catheterization. RUG analysis of each scaffold group at 3 m post-op demonstrated wide urethral calibers and preservation of organ continuity at the original implantation site similar to pre-operative and control features with no evidence of contrast extravasation, strictures, fistulas, or stone formation (**Figure 3**). Gross tissue evaluations revealed host tissue ingrowth spanning the entire area of the original implantation site in animals receiving silk fibroin grafts with negligible contraction observed between the proximal/distal or lateral marking sutures (**Figure 2D**); similar results were obtained with SIS scaffolds. In addition, macroscopic examination of the upper urinary tract showed no signs of hydronephrosis or renal anomalies in controls or animals implanted with silk fibroin or SIS scaffolds. These observations are consistent with the lack of significant alterations in serum creatinine levels in all experimental groups over the course of the study (data not shown) suggestive of normal kidney function.

**Figure 4:**
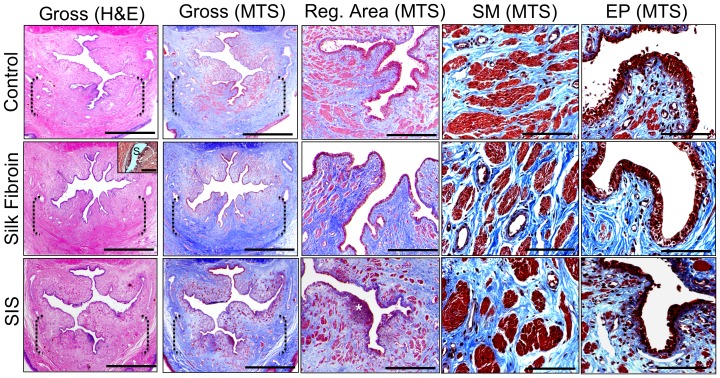
Histological evaluations (H&E and MTS analyses) of urethral tissue regeneration in control and implant groups following 3 m post-op. [1^st^ and 2^nd^ columns] Photomicrographs of gross penile cross-sections in proximal regions of tissue repair. Scale bars = 3 mm. Inset: residual silk fibroin matrix fragment (S), scale bar = 100 µm. Brackets represent sites of original scaffold implantation or control urethrotomy. [3^rd^ column] Magnification of global tissue regeneration bracketed in 2^nd^ column. Scale bars = 600 µm. (*) = aggregate of mononuclear cells indicative of chronic inflammation. [4^th^ and 5^th^ columns] Magnified *de novo* smooth muscle (SM) and epithelial (EP) tissue formation displayed in 3^rd^ column. Scale bars = 200 µm.

Global histological examinations (H&E and MTS analyses) at 3 m post-op demonstrated that in both implant groups there was prominent ingrowth of connective tissue from the host urethral wall along the entire longitudinal axis of the original implantation site (**Figure 4**). Cross-sectional organization throughout the *de novo* urethral wall in each scaffold group resembled control tissue architecture with distinct tissue compartments consisting of a luminal, multi-layered epithelium, an extra-cellular matrix (ECM)-rich lamina propria, and an outer smooth muscle layer. Comparable degrees of scaffold degradation were qualitatively observed in both implant groups following the 3 m study period with minute residual matrix fragments dispersed throughout the regenerated tissue (**Figure 4** **, insert**).

**Figure 5:**
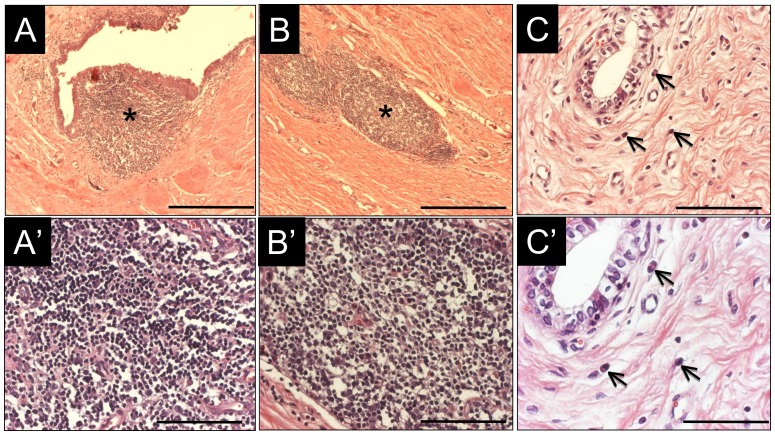
Inflammatory responses elicited by scaffold groups. Photomicrographs of acute and chronic inflammatory reactions in H&E-stained proximal regions of tissue repair following 3 m post-op. Mobilized follicular aggregates of mononuclear cell infiltrates [chronic inflammation, denoted by (*)] present in sub-epithelial [A, A′ (magnified view)] and lamina propria [B, B′ (magnified view)] regions of *de novo* tissue supported by SIS implants. [C, C′ (magnified view)] Eosinophil granulocytes (acute inflammation, denoted by arrows) present in *de novo* tissue supported by silk fibroin scaffolds. [A, B], scale bars = 600 µm; [A′, B′, C], scale bars = 200 µm, [C′], scale bar = 100 µm.

MTS analysis demonstrated discrete areas of mild fibrosis within the lamina propria of the consolidated tissues supported by each matrix configuration (data not shown). However, SIS matrices were found to elicit chronic inflammatory reactions in the sub-epithelial (**Figure 5A, A′**) and lamina propria (**Figure 5B, B′**) regions of the *de novo* urethral tissue characterized by significant induction of mobilized follicular aggregates of mononuclear cell infiltrates (density, 2.0±0.8 aggregates/10 mm^2^ tissue area; aggregate diameter, 462±241 µm) in comparison to control (*p*<0.010) and silk fibroin groups (*p*<0.010). In contrast, occasional eosinophil granulocytes indicative of minimal acute inflammatory reactions were observed following silk fibroin scaffold implantation at 3 m post-op (**Figure 5C, C′**) with no evidence of mononuclear cell infiltrates. These immunogenic responses are similar to those previously detected in rodent models of bladder augmentation with silk fibroin biomaterials [39].

**Figure 6:**
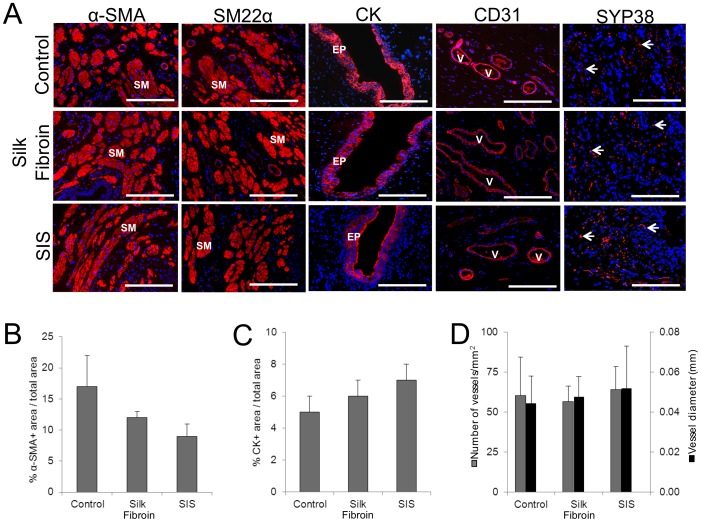
Immunohistochemical and histomorphometric assessments of urethral tissue regeneration in control and scaffold groups following 3-op. [A] Photomicrographs of protein expression of smooth muscle (SM) contractile markers (α-SMA and SM22α); epithelial (EP)-associated cytokeratins (CK); the endothelial marker, CD31; and the innervation marker, synaptophysin (SYP38) in proximal sites of tissue repair. V denotes vessels and arrows denote neuronal lineages. For all panels, respective marker expression is displayed in red (Cy3 labeling) and blue denotes DAPI nuclear counterstain. Scale bars in all panels = 200 µm. [B–D] Histomorphometric analysis of the extent of regenerated α-SMA+ smooth muscle bundles [B], CK+epithelium [C], and CD31+ vessels [D] present in the original surgical sites of control and scaffold groups.

IHC evaluations (**Figure 6A**) revealed α-SMA and SM22α contractile protein expression in the reconstituted smooth muscle layers supported by both scaffold groups as well as controls indicative of smooth muscle differentiation. Epithelial maturation in controls and throughout all regenerated tissues supported by silk fibroin and SIS matrices was also confirmed by robust CK expression. Histomorphometric analysis demonstrated statistically similar extents of α-SMA+ smooth muscle bundles (**Figure 6B**) and CK+ epithelium (**Figure 6C**) within the original surgical sites across all experimental conditions reflecting comparable degrees of smooth muscle and epithelial tissue regeneration. Evidence of *de novo* vascularization and innervation processes were also detected in each implant group. The number and diameter of vessels containing CD31+ endothelial cells within the original surgical sites were found to be statistically similar across all experimental conditions as determined by histomorphometric analysis (**Figure 6D**). In addition, neuronal lineages displaying prominent SYP38 protein expression indicative of synaptic transmission areas were also localized throughout the *de novo* urethra walls in both implant groups as well as in controls. These data demonstrate that silk fibroin scaffolds are capable of supporting regeneration of innervated, vascularized smooth muscle and epithelial tissues in a rabbit model of urethra repair.

## Conclusions

The results presented in this study detail the feasibility of bi-layer silk fibroin scaffolds to serve as acellular grafts for onlay urethroplasty. Performance comparisons with conventional SIS biomaterials demonstrated that silk fibroin matrices displayed superior biocompatibility, due to the absence of chronic inflammatory reactions, while promoting similar degrees of tissue regeneration as well as maintenance of urethral function following 3 m of implantation. Future studies will focus on long-term evaluations and efficacy assessments in animal models of urethral disease in order to ascertain the potential of silk fibroin scaffolds for clinical urethral reconstruction.
